# Acknowledgement to Reviewers of *Metabolites* in 2014

**DOI:** 10.3390/metabo5010001

**Published:** 2015-01-08

**Authors:** Metabolites Editorial Office

**Affiliations:** MDPI AG, Klybeckstrasse 64, CH-4057 Basel, Switzerland

The editors of *Metabolites* would like to express their sincere gratitude to the following reviewers for assessing manuscripts in 2014:

Abraham, Wolf-Rainer
Alric, Jean
Amann, Anton
Ammann, Anton
Appel, Jens
Arimura, Gen-Ichiro
Au, Kit Sing
Bais, Preeti
Bamba, Takeshi
Barlow, Chris
Bathe, Oliver F.
Bearden, Dan
Beauchamp, Jonathan
Beck, John J.
Bertrand, Samuel
Biasioli, Franco
Bielawski, J.
Bigger, Brian W.
Blasco, Hélène
Bos, Liewe
Boss, Paul K.
Bronze, Maria
Bruni, Paola
Budhu, Anuradha
Bundy, Jacob
Burcynski, Frank
Burnap, Robert
Buszewski, BogusÅ‚aw
Campanini, Barbara
Canbay, Ali
Cardol, Pierre
Chauvat, Franck
Chen, Chi
Conejero, Alberto
Cuperlovic-Culf, Miroslava
Cusido, Rosa M.
Czech, Christian
De Gennaro, Gianluigi
De Magalhães, João Pedro
Ding, Mingyu
Dirusso, Concetta C.
Dismukes, G. Charles
Dolferus, Rudy
Dragsted, Lars
Dunn, Warwick
Durnford, Dion
Edison, Arthur
Eghbalnia, Hamid R.
Elbahrawy, Mona
Eleftheriadis, Theodoros
Falkowski, Paul
Fang, Ye
Feist, Adam M.
Fendt, Sarah-Maria
Florencio, Francisco J.
Francois, Jean Marie
Freund, Dana
Frizzell, Norma
Gelb, Michael H.
Giordano, Mario
Gleissman, Helena
Glenn, Thomas C.
Gonçalves, Olivier
Green, David H.
Gronwald, Wofram
Guha, Manti
Gustafsson, Lars L.
Hagemann, Martin
Han, Jaehong
Hancock, Rob
Harmatz, Paul R.
Hasanuzzaman, Mirza
Hasunuma, Tomohisa
Herbig, Jens
Hippler, Michael
Hoene, Miriam
Holzhütter, Herrmann-georg
Horiuchi, Jun-ichi
Huege, Jan
Iorio, Egidio
Janfelt, Christian
Jareño-Esteban, José Javier
Jazwinski, S. Michal
Jeandet, Philippe
Jünger, Melanie
Karp, Peter D.
Kataoka, H.
Khoomrung, Sakda
Kiselev, K.V.
Kopriva, Stanislav
Ku, Kuo-Lung
Lang, Christine
Le Bihan, Thierry
Leung, Kit-Yi
Li, Jianfeng
Longo, Nicola
Macdonald, Jeffrey M.
Mahuran, Don J.
Marincola, Flaminia Cesare
Martin, François-Pierre J.
McCarty, Douglas M.
Mehta, Minesh P.
Mercolini, Laura
Mettler, Tabea
Mimmo, Tanja
Mimura, Tetsuro
Moenner, Michel
Moing, Annick
Moore, Mark
Morgan, John
Motohashi, Hozumi
Mundra, Piyush
Murabito, Ettore
Niehaus, Karsten
Niinemets, Ülo
Northen, Trent
Obata, Toshihiro
Oefner, Peter J.
Oikawa, Akira
Okazawa, Atsushi
Palazon, Javier
Pan, Min-Hsiun
Pańka, Dariusz
Passos, Fabiana
Peck, Dawn
Pedinti, Gopal
Perl, Thorsten
Phillips, Chris O.
Poupon, Erwan
Pulinilkunnil, Thomas
Puri, Basant
Puy, Hervé
Rabinowitz, Joshua D.
Ratcliffe, George
Richardson, Adam
Rigas, Pantelis G.
Roberts, Rachel M.
Rogers, Simon
Santonico, Marco
Sasayama, Takashi
Schäuble, Sascha
Schild, Detlev
Schroda, Michael
Schultz, Michael C.
Sharma, Kumar
Shinano, Takuro
Simeonidis, Evangelos
Smith, David
Splivallo, Richard
Steinmeyer, Juergen
Sterk, Peter
Stewart, M.F.
Stoelzel, Ulrich
St-Pierre, Julie
Styczynski, Mark
Sussman, Mike
Svatos, Ales
Takors, Ralf
Taymaz-Nikerel, Hilal
Thiel, Teresa
Toya, Yoshihiro
Treasaden, Ian H.
Van der Spoel, Aarnoud C.
Van Velzen, Ewoud J. J.
Vega, Jose
Viguera, Cristina
Vrhovsek, Urska
Wawrzyńska, Anna
Węgrzyn, Grzegorz
Wendell, Stacy Gelhaus
Wijburg, Frits A.
Wittmann, C.
Wood, Paul L.
Yamada, Tesshi
Yetukuri, Laxman
Yin, Huiyong
Yuneva, Mariia
Zrobek-Sokolnik, A.
Zwartkruis, Fried J.

